# Health Problems during Compulsory Military Service Predict Disability Retirement: A Register-Based Study on Secular Trends during 40 Years of Follow-Up

**DOI:** 10.1371/journal.pone.0159786

**Published:** 2016-08-17

**Authors:** Heikki Frilander, Tea Lallukka, Eira Viikari-Juntura, Markku Heliövaara, Svetlana Solovieva

**Affiliations:** 1 Centre of Expertise for Health and Work Ability, Finnish Institute of Occupational Health, Helsinki, Finland; 2 Disability Prevention Research Centre, Finnish Institute of Occupational Health, Helsinki, Finland; 3 Department of Health, National Institute for Health and Welfare, Helsinki, Finland; Universidade Federal do Rio de Janeiro, BRAZIL

## Abstract

Disability retirement causes a significant burden on the society and affects the well-being of individuals. Early health problems as determinants of disability retirement have received little attention. The objective was to study, whether interrupting compulsory military service is an early indicator of disability retirement among Finnish men and whether seeking medical advice during military service increases the risk of all-cause disability retirement and disability retirement due to mental disorders and musculoskeletal diseases. We also looked at secular trends in these associations. We examined a nationally representative sample of 2069 men, who had entered military service during 1967–1996. We linked military service health records with cause-specific register data on disability retirement from 1968 to 2008. Secular trends were explored in three service time strata. We used the Cox regression model to estimate proportional hazard ratios and their 95% confidence intervals. During the follow-up time altogether 140 (6.8%) men retired due to disability, mental disorders being the most common cause. The men who interrupted service had a remarkably higher cumulative incidence of disability retirement (18.9%). The associations between seeking medical advice during military service and all-cause disability retirement were similar across the three service time cohorts (overall hazard ratio 1.40 per one standard deviation of the number of visits; 95% confidence interval 1.26–1.56). Visits due to mental problems predicted disability retirement due to mental disorders in the men who served between 1987 and 1996 and a tendency for a similar cause-specific association was seen for musculoskeletal diseases in the men who served in 1967–1976. In conclusion, health problems—in particular mental problems—during late adolescence are strong determinants of disability retirement. Call-up examinations and military service provide access to the entire age cohort of men, where persons at risk for work disability can be identified and early preventive measures initiated.

## Introduction

Disability retirement leading to early exit from working life is a major concern for the society in most developed countries, not least due to the economic burden the absence from productive working life poses. In Finland, the prevalence of disability retirement among working age people is 6.8% [1] Musculoskeletal diseases and mental disorders have remained the two leading causes of disability retirement in the Western countries for decades [2]. Incidence of disability retirement due to mental disorders increased in the late 1980s in Finland, leading to receivers of these pensions being the largest group among the disability retired [1, 3]. Similar trends and similar patterns across genders have been seen also in other European countries [2, 4, 5].

Numerous studies have looked at the predictors of disability retirement, focusing mainly at risk factors occurring in the middle aged [6]. Earlier adulthood predictors of disability retirement have received less attention [7]. Mental and musculoskeletal disorders have been reported to co-occur frequently in the general population and have a synergistic effect on work disability [8, 9].

Compulsory military service with several health check-ups offer a unique opportunity to identify early indicators for later life events. The Finnish Defence Forces are based on universal male conscription, therefore the entire male age class undergoes a health examination during call-up and up to 80% of the young men enter military service. The proportion of men who have been exempted at call-up has been quite stable until lately, but the proportion of those who have interrupted their military service has increased substantially during the last decades [10, 11]. The length of military service was 8 to 11 months until 1993 and is thereafter 6 to 12 months. The service time of conscripts being trained as officers, non-commissioned officers and for the more demanding duties of rank personnel is longer than for those trained for rank duties. The conscripts spend their first two months of military service in basic training, which with regard to the content of training is basically the same for all of them. After this period the training is more diversified. It is common to seek medical advice during compulsory military service and the mean number of health care consultations per conscript during service has more than doubled from below four to over eight visits since the late 60s [12].

Very few studies have examined the impact of seeking medical advice in youth or adolescence on adult disability pensions. A Swedish study reported that being sometimes absent from school at the age 16 predicted adult sickness absence only in women, not in men [13]. Although both musculoskeletal and mental problems manifest already in the youth [14–16], little is known about care-seeking in youth and later disability.

The aims of this register-linked study were to examine, whether interrupting compulsory military service is an early indicator of disability retirement among Finnish men and whether seeking medical advice during military service increases the risk of all-cause disability retirement and disability retirement due to mental disorders and musculoskeletal diseases. In addition, we looked at secular trends in these associations in three service time periods covering 30 years.

## Materials and Methods

### Study population

**Fig 1:**
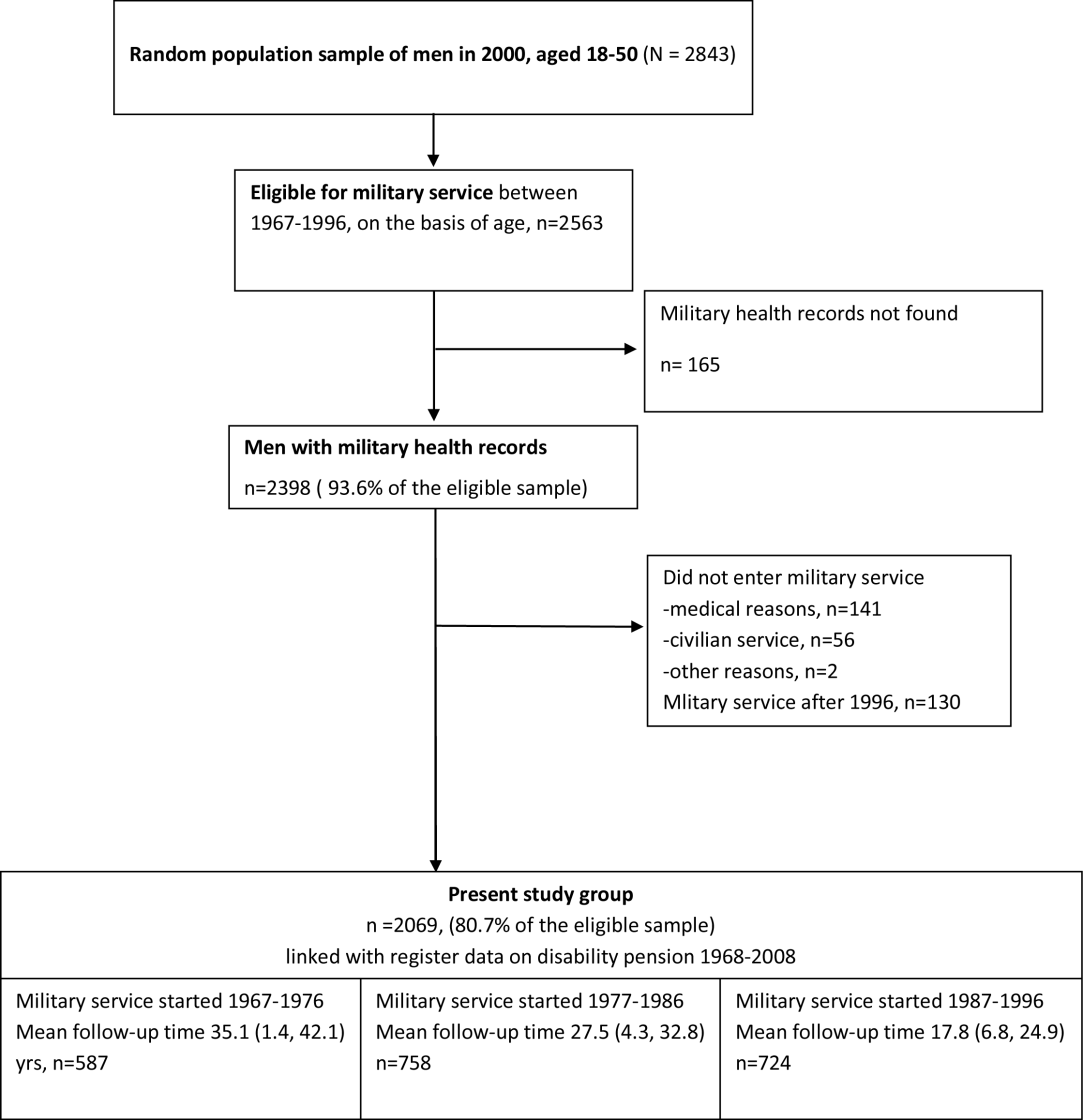
Flowchart of the Present Study Sample.

The current study used a longitudinal design (Fig 1). A random nationally representative sample of 2843 men aged 18–50 years was drawn from the population register by a two-stage cluster sampling as a part of the Health 2000 Study. The comprehensive Health 2000 study was carried out in Finland during 2000–2001 in order to achieve an overall view of the population’s health [17, 18]. The participants in the Health 2000 Study have given written informed consent for using their data in studies on health, various diseases and their determinants, including linkage with a number of registers, e.g. those containing information on disability retirement.

For this study eligible were all men who belonged to the Health 2000 sample and were eligible for military service during 1967–1996 (n = 2563, i.e. born in 1948–1978). The military records of the men were searched from the register of the Finnish Defence Forces (Musculoskeletal Disorders in Military Service Study, MSDs@Mil). Military health records were not available for 165 (6.4%) men. Of the 2563 eligible men 199 (7.0%) did not enter military service, mostly due to medical reasons (n = 141) and 130 (5.0%) men entered after 1996.

After exclusion the study sample comprised of 2069 (80.7% of the eligible sample). The sample was stratified into three groups on the basis of the service year: 1967–1976 (n = 587), 1977–1986 (n = 758), and 1987–1996 (n = 724).

### Ethical approval

The Section for Epidemiology and Public Health of the Ethics Committee of the Hospital District of Helsinki and Uusimaa approved the Health 2000 Study and The Ethics Committee of the Finnish Institute of Occupational Health further approved the MSDs@Mil study.

### Seeking medical advice during compulsory military service

Data from military medical files were collected from the registers of the Finnish Defence Forces using personal identity numbers. The Finnish Defence Forces gave permission to use these data for the purposes of this study. Detailed information of each visit due to musculoskeletal or mental problems to the military healthcare units was coded from the military service medical records. Also all other consultations to military healthcare due to any reason were identified and the reasons coded (cardiac, respiratory, neurological, infectious, dermatological, other reason). The data collection methods have been described in more detail elsewhere [12]. The total number of visits to military healthcare due to any reason, and those due to mental or MSD-related problems per service month was calculated.

### Disability retirement

Data on disability retirement were obtained from the national registers of the Finnish Centre for Pensions and The Social Insurance Institution of Finland. Complete information was provided on all retirement events and their main and secondary diagnoses. All disability pensions granted before 1 January 2009 were linked to the MSDs@Mil data using personal identity numbers. In Finland, people with a chronic illness, disability, or injury that has been verified by a physician with a medical certificate and evaluated as causing considerable and long-lasting decreased work ability are entitled to a part-time or full-time disability pension [19]. The diagnoses of chronic illnesses in the national pension register were classified according to The International Statistical Classification of Diseases and Related Health Problems, Eight, Ninth and-Tenth Revisions (ICD-8, -9 and -10, Finnish versions of ICD-classifications 1969, 1987, 1996).

The main outcome variable was all-cause disability retirement, which comprised all incident permanent, temporary or part-time disability retirements for any disease at any time between 1 January 1968 and 1 January 2009. We also looked at cause-specific disability retirement due to mental disorders and musculoskeletal diseases.

### Covariates

The date of entry in military service and length of service were derived from military files. The year of entry was considered as the year of service and used as a covariate in the analyses.

### Statistical analyses

The collected register data were anonymized and de-identified prior to the analyses.

Descriptive statistics were presented as proportions and mean with 95% confidence intervals (95% CI) or median and interquartile interval (IQI).

Associations between seeking medical advice (visits due to health problems per service month) during military service and disability retirement were estimated using Cox proportional hazard regression analysis and were reported as hazard ratios (HRs) with 95% confidence intervals (95% CIs). The distributions of the visits due to any health problem, or due to mental or musculoskeletal problems were very skewed and varied by year of service as well as service length. Thus the service year adjusted Z-scores were calculated for each type of visits and used in the analyses. All analyses were performed for each service time stratum. The pooled HRs were estimated using the Mantel-Haenszel method.

The follow-up of each subject started from the first day after completing or interrupting military service and continued until the beginning of disability pension or other pension, death, or end of follow-up (01.01.2009), whichever came first. Respondents who retired due to other reasons (i.e. age, n = 3) or died during follow-up (n = 39) were censored. The follow-up time was counted in days.

To investigate whether the associations depended on the length of follow-up time, we conducted the analyses in four follow-up time-strata: 0 to 4745 days (0–13.0 years), 4746 to 8395 days (13.1–23.0 years), 8396 to 12045 days (23.1–33.0 years) and 12046 to 15330 days (> 33.0 years) of follow up.

The proportional hazard assumption was assessed visually through inspection of the log-log hazards plots on all predictors, and was found to be satisfactory. All continuous predictors were categorised prior to the assessment.

In additional analyses we looked whether pre-military BMI confounded the associations. Kaplan–Meier survival curves were plotted to show the time to disability retirement for each category of the determinant variable, i.e. visits due to musculoskeletal and mental problems. An interaction between two risk factors on additive scale is present, if the relative excess risk due to interaction (RERI) is not equal to zero. The RERI >0 will indicate synergism between two risk factors. To test whether visits due to mental and musculoskeletal problems synergistically associated with disability retirement, the relative excess risk due to interaction between visits due to mental and musculoskeletal problems was calculated [20].

Statistical analyses were performed using SAS 9.4.

## Results

### Characteristics of the study participants

The mean age of the 2069 men who composed our study sample was 37.0 years (95% CI 36.7, 37.4). The mean age at the time of service was 20.05 years (20.0, 20.1). Men for whom military records were not found were older (mean age 38.9 years, 95%CI 37.5, 40.3), while those who did not enter military service were younger (mean age 34.4 years, 95%CI 33.2, 35.6) than those who were included into the study.

The average follow-up time was 26.2 (25.9, 26.6) years, being 35.1, 27.5 and 17.8 years for the men entering military service in 1967–1976, 1977–1986 and 1987–1996, respectively (Fig 1). Of the 2069 men who entered the military service, 111 (5.4%) did not complete their service, mainly due to medical reasons (n = 91, 82.0%). The median length of the service for men who interrupted their service was 54 days (interquartile range (IQR) 16–120 days).

In the random sample of eligible men the cumulative incidence of all-cause disability retirement was 8.8% and in the study group 6.8%. The cumulative incidence of all -cause disability retirement and disability retirement due to mental disorders were remarkably higher among men who did not enter military service due to medical reasons compared to those who entered (Table 1).

**Table 1:** Cumulative incidence (%) of disability retirement in the random population sample.

	Eligible for military service 1967–1996		Excluded from study group								Study group	
			*Military health records not found*		*Did not start military service due to medical reasons*		*Civilian service*		*Served after 1996*			
	*n = 2563*		*n = 165*		*n = 141*		*n = 56*		*n = 130*		*n = 2069*	
Disability retirement	n	% (95% CI)	n	% (95% CI)	n	% (95% CI)	n	% (95%CI)	n	% (95% CI)	n	% (95% CI)
All-cause	227	8.8 (7.8–10.0)	19	11.5 (7.4–17.4)	60	43.0 (34.7–50.8)	5	8.9 (3.5–19.7)	3	2.3 (0.5–6.9)	140	6.8 (5.8–7.9)
Mental disorders	111	4.3 (3.6–5.2)	5	3.0 (1.1–7.1)	39	27.7 (20.9–35.6)	2	3.6 (0.3–12.8)	3	2.3 (0.5–6.9)	62	3.0 (2.3–3.8)
Musculoskeletal diseases	35	1.4 (1.0–1.9)	5	3.0 (1.1–7.1)	1	0.7; (0.01–4.0)	0	0.0	0	0.0	29	1.4 (1.0–2.0)

n, number; CI, confidence interval.

### Seeking medical advice

Most of the men visited military healthcare at least once during their service (S1 Table). The proportion of men with at least one visit due to a mental problem alone or due to both mental and musculoskeletal problems tripled with time. The median number of visits to military healthcare due to any reason was 4 (interquartile interval (IQI) 2–8). There was a distinct increase in the number of visits among those men, who started their military service during 1987–1996 (median 6, IQI 3–11). Half of the men had at least one healthcare visit due to any reason per each two months of service (S2 Table). For the three service time strata the median numbers were 0.3, 0.4 and 0.7 visits per service month, accordingly. The men who interrupted their military service had markedly higher numbers of visits relative to the length of service than those who completed the service.

### Cumulative incidence of outcomes

The cumulative incidence of disability pensions due to mental disorders was 3.0%, and that of musculoskeletal diseases 1.4% (Table 1). The vast majority (95%) of disability pensions was granted as full-time and permanent. Table 2 presents the proportions of those who were awarded disability pension by the service period as well as by the length of follow-up. Disability retirement due to mental disorders was awarded typically after 13 years of follow-up (mean time to retirement 21.3; 95% CI 19.1–23.6 years), while disability retirement due to musculoskeletal diseases was awarded after 23 years of follow-up (mean time to retirement 26.7; 95% CI 24.1–29.2 years). Both all-cause disability retirement and disability retirement due to mental disorders were especially prevalent among the men, who had interrupted their military service as compared with those who completed the service (Table 3).

**Table 2:** Cumulative incidence (%) of disability retirement in the study group by the military service period and the length of follow-up.

	**Military service period**							
	*1967–1976*		*1977–1986*		*1987–1996*			
	*n = 587*		*n = 758*		*n = 724*			
**Disability retirement**	n	% (95% CI)	n	% (95% CI)	n	% (95% CI)		
all-cause	79	13.5 (10.1–16.5)	44	5.8 (4.3–7.7)	17	2.2 (1.4–3.7)		
due to mental disorders	27	4.6 (3.1–6.6)	24	3.2 (2.0–4.7)	10	1.5 (0.7–2.5)		
due to musculoskeletal diseases	17	2.9 (1.7–4.6)	10	1.3 (0.6–2.4)	2	0.3 (0.03–1.0)		
	**Length of follow-up**							
	*0–13*.*0 years*		*13*.*1–23*.*0 years*		*23*.*1–33*.*0 years*		*> 33*.*0 years*	
	*n = 2069*		*n = 2042*		*n = 1268*		*n = 483*	
**Disability retirement**	n	% (95% CI)	n	% (95% CI)	n	% (95% CI)	n	% (95% CI)
all-cause	19	0.9 (0.6–1.4)	40	2.0 (1.4–2.7)	57	4.5 (3.4–5.8)	24	5.0 (3.2–7.3)
due to mental disorders	12	0.6 (0.3–1.0)	25	1.2 (0.8–1.8)	19	1.5 (0.9–2.3)	6	1.2 (0.5–2.7)
due to musculoskeletal diseases	1	0.001	6	0.3 (0.1–0.6)	18	1.4 (0.8–2.2)	4	0.8 (0.2–2.1)

n, number; CI, confidence interval.

**Table 3:** Cumulative incidence (%) of disability retirement by completeness of military service, in three service time strata.

	Did not complete military service (n = 111)								Completed military service (n = 1958)							
	1967–1976		1977–1986		1987–1996		All, 1967–1996		1967–1976		1977–1986		1987–1996		All, 1967–1996	
	*n = 24*		*n = 26*		*n = 61*		*n = 111*		*n = 563*		*n = 732*		*n = 663*		*n = 1958*	
Disability retirement	*n*	*%*	*n*	*%*	*n*	*%*	*n*	*%*	*n*	*%*	*n*	*%*	*n*	*%*	*n*	*%*
		*(95% CI)*		*(95% CI)*		*(95% CI)*		*(95% CI)*		*(95% CI)*		*(95% CI)*		*(95% CI)*		*(95% CI)*
all-cause	10	41.7	4	15.4	7	11.5	21	18.9	69	12.3	40	5.5	9	1.4	118	6.0
		(24.6–61.2)		(5.5–34.2)		(5.4–22.1)		(12.7–27.3)		(9.8–15.2)		(4.0–7.4)		(0.7–2.6)		(5.1–7.2)
due to mental disorders	5	20.8	3	11.5	4	6.6	12	10.8	22	3.9	21	2.9	6	0.9	49	2.5
		(8.8–40.9)		(3.2–29.8)		(2.1–16.1)		(6.2–18.1)		(2.6–5.9)		(1.9–4.4)		(0.4–2.0)		(1.9–3.3)
due to musculo-skeletal diseases	2	8.3	1	3.8		0	3	2.7	15	2.7	9	1.2	2	0.3	26	1.3
		(1.2–27.0)		(0.0–20,5)		(0.0)		(0.6–8.0)		(1.6–4.4)		(0.6–2.4)		(0.01–1.2)		(0.9–2.0)

n, number; CI, confidence interval.

### Associations between seeking medical advice during military service and disability retirement

Seeking frequently medical advice during military service was associated with all-cause disability retirement in each of the three service time strata, the association being strongest for the latest cohort (HR 1.72 for one visit per service month during military service, Table 4). Seeking medical advice due to mental or musculoskeletal problems was associated with all-cause disability in the earliest and especially in the latest service stratum. Furthermore, in the latest stratum visits due to mental problems and those due to musculoskeletal problems were both associated with a 1.4-fold higher hazard of disability retirement due to mental disorders. Visits due to any health problems were associated with disability retirement due to musculoskeletal diseases in the earliest service time stratum, while for the cause-specific visits only a tendency was seen.

**Table 4:** Seeking care (visits per service months) during military service and disability retirement by the service time strata.

	1967–1976		1977–1986		1987–1996		All, 1967–1996	
	n = 587		n = 758		n = 724		n = 2069	
	*HR*^*a*^	*95% CI*	*HR*^*a*^	*95% CI*	*HR*^*a*^	*95% CI*	*HR*^*a*^	*95% CI*
**All-cause disability retirement**								
Visits due to any problem^b^	1.28	1.09–1.49	1.42	1.13–1.79	1.72	1.43–2.07	1.40	1.26–1.56
Visits due to mental problems^c^	1.21	1.07–1.36	0.95	0.67–1.36	1.34	1.09–1.65	1.17	1.06–1.29
Visits due to musculoskeletal problems^d^	1.25	1.05–1.47	1.14	0.89–1.47	1.49	1.23–1.81	1.28	1.14–1.44
**Disability retirement due to mental disorders**								
Visits due to any problem^b^	1.11	0.78–1.58	1.56	1.17–2.06	1.74	1.37–2.21	1.44	1.23–1.68
Visits due to mental problems^c^	0.96	0.59–1.58	1.04	0.74–1.47	1.41	1.13–1.77	1.14	0.97–1.34
Visits due to musculoskeletal problems^d^	0.65	0.34–1.26	1.20	0.88–1.64	1.40	1.05–1.86	1.12	0.89–1.40
**Disability retirement due to musculoskeletal diseases**								
Visits due to any problem^b^	1.59	1.27–2.00	0.91	0.55–2.34	-	-	1.42	1.14–1.78
Visits due to mental problems^c^	1.14	0.84–1.55	-	-	-	-	1.05	0.77–1.43
Visits due to musculoskeletal problems^d^	1.33	0.96–1.83	1.12	0.65–1.93	-	-	1.23	0.93–1.61

n, number; HR, hazard ratio; CI, confidence interval.

^a^ per one SD in the number of visits

^b^one SD corresponds to 0.6 visits per service month for military service during 1967–1986 and 1.2 visits per month for military service during 1987–1996

^c^one SD corresponds to 0.1 visits per service month for military service during 1967–1976, 0.3 visits per service month for military service during 1977–1986 and 0.7 visits per month for military service during 1987–1996

^d^one SD corresponds to 0.2 visits per service month for military service during 1967–1976, 0.4 visits per service month for military service during 1977–1986 and 0.8 visits per month for military service during 1987–1996.

Analyses by the length of follow-up showed that seeking medical advice during military service was associated with disability retirement due to mental disorders after 13–23 years of follow-up, while the association with disability retirement due to musculoskeletal diseases was seen only after 23 years of follow-up (Table 5). Having sought medical advice due to mental problems showed a tendency for an association with disability retirement due to mental disorders after 13–23 years of follow-up. An additional analysis for the three service time strata showed that this association was statistically significant (HR 1.52, 95% CI 1.16, 2.01) for the men who served in 1987–1996. Finally, further adjustment for pre-military BMI did not affect any of the observed associations.

**Table 5:** Seeking care (visits per service months) during military service and disability retirement by the length of follow-up time strata.

	0–13.0 years^a^		13.1–23.0 years^b^		23.1–33.0 years^c^		> 33.0 years^d^	
	n = 2069		n = 2042		n = 1268		n = 483	
	*HR*^*e*^	*95% CI*	*HR*^*e*^	*95% CI*	*HR*^*e*^	*95% CI*	*HR*^*e*^	*95% CI*
**All-cause disability retirement**								
Visits due to any problem^f^	1.34	0.96–1.87	1.54	1.29–1.84	1.42	1.21–1.68	1.18	0.85–1.65
Visits due to mental problems^g^	1.17	0.88–1.56	1.14	0.93–1.40	1.21	1.07–1.37	1.08	0.72–1.63
Visits due to musculoskeletal problems^h^	1.17	0.80–1.70	1.27	1.04–1.59	1.35	1.14–1.60	1.11	0.75–1.64
**Disability retirement due to mental disorders**								
Visits due to any problem	1.42	0.96–2.12	1.60	1.30–1.97	1.39	1.03–1.87	0.13	0.01–1.50
Visits due to mental problems	1.24	0.92–1.68	1.20	0.97–1.46	0.96	0.56–1.64	-	-
Visits due to musculoskeletal problems	1.17	0.72–1.90	1.20	0.90–1.62	1.04	0.68–1.61	-	-
**Disability retirement due to musculoskeletal diseases**								
Visits due to any problem	^-^	^-^	1.20	0.62–2.33	1.43	1.08–1.91	1.69	1.12–2.54
Visits due to mental problems	-	-	-	-	1.11	0.82–1.51	-	-
Visits due to musculoskeletal problems	-	-	1.05	0.49–2.25	1.29	0.94–1.77	1.20	0.51–2.83

n, number; HR, hazard ratio; CI, confidence interval.^a^corresponds to 0 up to 4745 days

^b^corresponds 4746 up to 8395 days

^c^corresponds 8396 up to 12045 days

^d^corresponds to > 12045 days

^e^ per one SD in the number of visits

^f^one SD corresponds to 1.4 visits per service month

^g^one SD corresponds to 1 visits per service month

^h^one SD corresponds to 1.8 visit per service month.

**Fig 2:**
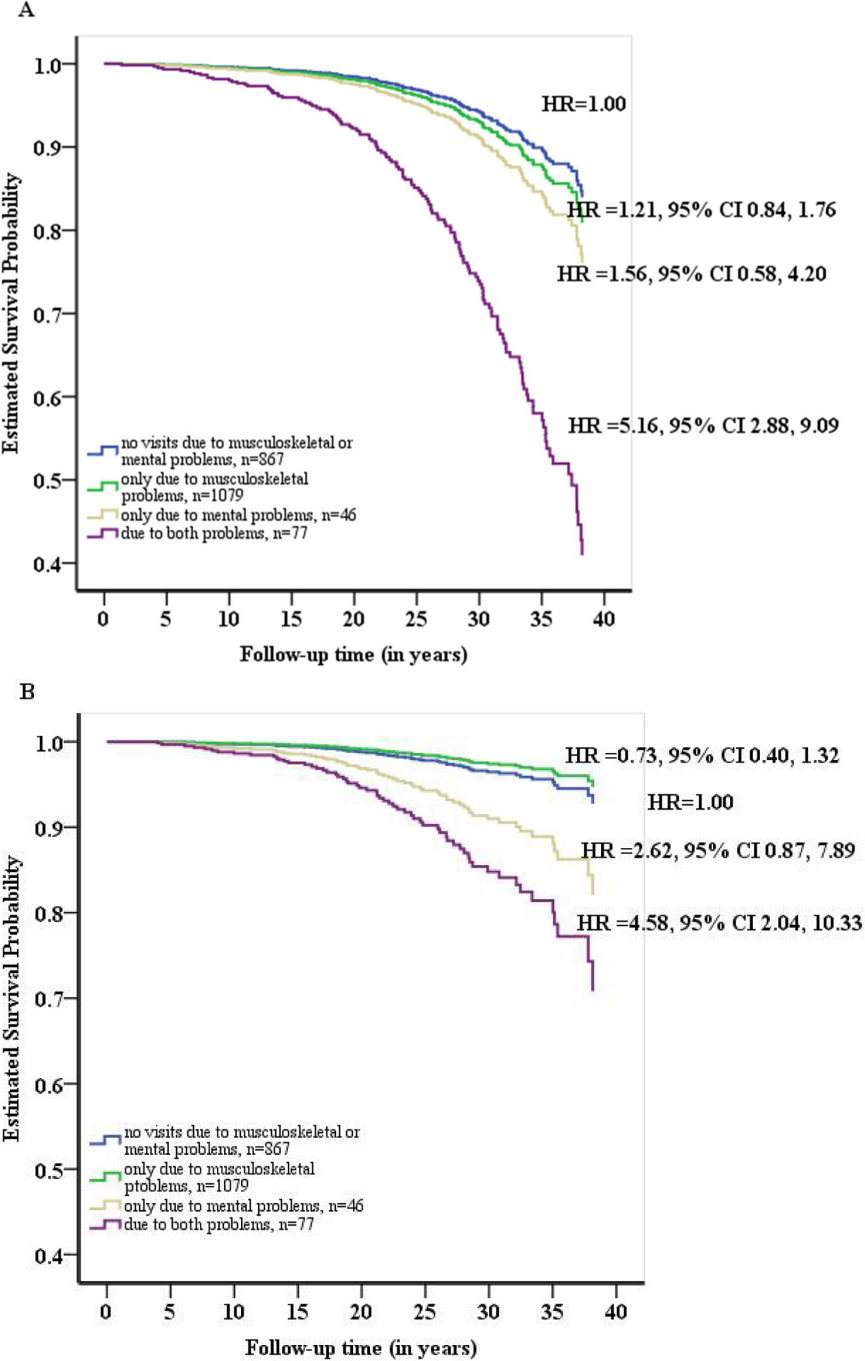
**Cumulative survival curves for the incidence of a) all-cause disability retirement and b) disability retirement due to mental disorders, for visits due to musculoskeletal and mental problems.** HR = hazard ratio, adjusted for year of service.

The Kaplan-Meier survival curves of disability retirement for men who had sought medical advice due to musculoskeletal or mental problems or both during military service are plotted in Fig 2. Seeking medical advice due to mental but not musculoskeletal problems decreased the time to all-cause disability retirement, though not statistically significantly due to a low number of events (Fig 2A). Seeking medical advice due to both musculoskeletal and mental problems showed a pronounced effect on the time to all-cause disability retirement (HR 5.16, 95% CI 2.88–9.09) compared to not seeking medical advice due to either of these problems. The relative excess risk due to their interaction was statistically significant (RERI 3.34, 95% CI 1.16–5.52). Seeking medical advice due to both mental and musculoskeletal problems was statistically significantly associated with disability retirement also due to mental disorders (HR 4.58, 95% CI 2.04–10.33), although the relative excess risk due to interaction was not statistically significant (Fig 2B).

## Discussion

In our register-based study we found that seeking medical advice during compulsory military service was associated with all-cause disability retirement as well as disability retirement due to mental disorders and musculoskeletal diseases. The associations between seeking medical advice and all-cause disability retirement were similar across the three service time cohorts. However, visits due to mental problems predicted disability retirement due to mental disorders in the youngest cohort and a tendency for a similar cause-specific association was seen for musculoskeletal diseases in the oldest cohort. The men who interrupted service had a higher cumulative incidence of disability retirement, especially due to mental disorders, compared with those who completed military service.

We observed a cause-specific association between visits due to mental problems and disability retirement due to mental disorders with a relatively short latency in the most recent cohort. Since the prevalence of disability retirement due to mental disorders was especially high among those who did not enter or interrupted their military service, our findings altogether indicate that persons who are at risk of disability retirement due to mental disorders at a relatively young age can be identified at call-up examinations and during military service. These findings coincide with the time period when the rate of disability retirement due to mental disorders started to rise in Finland [3, 21, 22].

Common mental disorders among the middle-aged predict both all-cause disability retirement and disability retirement due to mental disorders. [23–25] Due to a lack of studies on early determinants of disability retirement our results are not well comparable with those from previous studies. However, our findings are in line with the results from a Swedish study reporting that receiving a psychiatric diagnosis at conscription predicted disability retirement due to mental and also other disorders [7].

We saw an association between visits due to any problem and disability retirement due musculoskeletal diseases in the oldest cohort. The long latency time may be a reason why we did not observe statistically significant cause-specific associations for musculoskeletal diseases.

Co-occurring mental and musculoskeletal problems have been reported to involve a particularly high risk of future work disability among adult working populations [8, 9]. Indeed, we found a synergetic interaction between visits due to both musculoskeletal and mental problems and all-cause disability retirement, which was clearly higher than the earlier reported ones.

Several secular changes occurred in Finland during the follow-up of our study that may influence the disability retirement rate. First, treatment practices have evolved, i.e. non-specific back pain is no longer treated with rest. Second, in the early nineties there was a deep economic recession in Finland that may have increased the proneness to seek disability retirement. As a result, the relative share of individuals receiving a disability pension due to mental disorders and musculoskeletal diseases has changed during the follow-up. For example, the proportion of male disability pension receivers with mental disorders has increased from 32% in 1996 to 43% in 2008, while there has been a decrease from 27% to 22% in receivers of disability pension due to musculoskeletal diseases [22].

The three service time periods covered 30 years. At the same time the body weight of conscripts has increased and the physical fitness has decreased [26]. Since the physical requirements of military service have not essentially changed, the physical capacity of the conscripts may have reduced to meet the requirements of military service towards the end of our follow-up time. The increasing interruptions of service and the higher frequency of visits may be consequences of this development [12]. However, seeking frequently medical advice during military service may not reflect only health problems, but can also indicate inability to adapt to exceptional circumstances.

The present study has several strengths. First, we used a representative population sample, providing a unique opportunity to link military service records from 1967 to 1996 with register data on disability retirement from 1968 to 2008. Due to the universal conscription all men are obliged to attend the call-up examination, therefore military medical records could be obtained for almost all men in the sample. The study design and the complete data allowed us to explore the association between objectively measured early health indicators and disability retirement across a wide time span and with different lengths of follow-up. Furthermore, the skewed distribution of the number healthcare visits during military service and different lengths of service time as well as the increasing frequency of visits with time were taken into consideration in the analysis. With caution, our findings on the associations of early health problems with disability retirement can be generalised to countries with universal conscription and a similar social security system.

However, when interpreting the findings of the current study, some issues should be taken into consideration. For reasons not known to us many medical records for those born in 1949 were not found, resulting in an underrepresentation of those who served in the late 60s. This may have affected the statistical power to detect cause-specific associations for musculoskeletal diseases. Since our study population was sampled in 2000 and only those alive at the time of sampling formed the study base, health-based selection may have diluted the observed associations for the two earlier cohorts. However, there was no selection according to the outcomes of our study. Because all our participants were male, we were unable to explore gender differences. Due to a relatively small overall sample size and particularly the small size of the stratum with a long follow-up time we had limited power to study cause-specific associations. Nonetheless, since our main results regarding all-cause disability retirement were all statistically significant, it is unlikely that our study suffered from low statistical power. Furthermore, the selected analytical methods were suitable to examine a rare event.

Even if we were able to control for the length of military service, we could not take into consideration whether the type of training during the military service confounded the observed associations. We did not include the educational level or the socio-economic status of our participants or their parents because determinants of health problems at a young age were beyond the scope of our study. We acknowledge that we had limited data on potential confounders, therefore residual confounding cannot be ruled out.

## Conclusions

In conclusion, our findings indicate that health problems, in particular mental problems, during late adolescence are strong determinants of disability retirement. Our findings are of particular importance given the need to reverse the high rate of disability retirement due to mental disorders in Finland and other European countries. Call-up examinations and military service provide access to the entire age cohort of men, where persons at risk for work disability can be identified and early preventive measures initiated. Future studies should address similar research questions in women. For example, in countries with occupational health services with high coverage early health problems can be identified and their potential effect on future work disability assessed.

## Supporting Information

S1 Table Proportion (%) of subjects with healthcare visits during military service by the service period. (DOCX)

S2 Table Median and interquartile range of healthcare visits during military service relative to the service length (visits per service month). (DOCX)
