# Supplementary material for: Health Problems during Compulsory Military Service Predict Disability Retirement: A Register-Based Study on Secular Trends during 40 Years of Follow-Up
Source: PLoS One. 2016 Aug 17;11(8):e0159786. doi: 10.1371/journal.pone.0159786 (PMC4988709; doi:10.1371/journal.pone.0159786)
Supplement: S1 Table — (DOCX) [file pone.0159786.s001.docx]

**S1 Table. Proportion (%) of subjects with healthcare visits during military service by the service period.**

|  | **1967-1976 (n=587)** | | **1977-1986 (n=758)** | | **1987-1996 (n=724)** | | **1967-1996 (n=2069)** | |
| --- | --- | --- | --- | --- | --- | --- | --- | --- |
|  | *n* | *% (95% CI)* | *n* | *% (95% CI)* | *n* | *% (95% CI)* | *n* | *% (95% CI)* |
| Due to any reason | 490 | 83.5 (80.3-86.3) | 676 | 89.3 (86.8-91.2) | 677 | 93.5 (91.5-95.1) | 1843 | 89.1 (87.7-90.4) |
| Due to musculoskeletal problems | 273 | 46.5 (42.5-50.6) | 411 | 54.2 (50.7-57.7) | 472 | 65.2 (61.7-68.6) | 1156 | 55.9 (53.7-58.0) |
| Due to mental problems | 19 | 3.2 (2.1-5.0) | 35 | 4.6 (3.3-6.4) | 69 | 9.5 (7.8-11.9) | 123 | 5.9 (3.0-7.1) |
| Due to both musculoskeletal and to mental problems | 12 | 2.0 (1.1-3.6) | 20 | 2.6 (1.7-4.1) | 45 | 6.2 (4.7-8.2) | 77 | 3.7 (3.0-4.6) |
