# Supplementary material for: Health Problems during Compulsory Military Service Predict Disability Retirement: A Register-Based Study on Secular Trends during 40 Years of Follow-Up
Source: PLoS One. 2016 Aug 17;11(8):e0159786. doi: 10.1371/journal.pone.0159786 (PMC4988709; doi:10.1371/journal.pone.0159786)
Supplement: S2 Table — (DOCX) [file pone.0159786.s002.docx]

**S2 Table 2. Median and interquartile range of healthcare visits during military service relative to the service length (visits per service month).**

|  | **1967-1976** | **1977-1986** | **1987-1996** | **1967-1996** |
| --- | --- | --- | --- | --- |
|  | *M (IQR)* | *M (IQR)* | *M (IQR)* | *M (IQR)* |
| **All** | **n=587** | **n=758** | **n=724** | **n=2069** |
| Number of any garrison visits per service month | 0.3 (0.1-0.6) | 0.4 (0.2-0.8) | 0.7 (0.4-1.4) | 0.5 (0.2-0.9) |
| Number of visits due to mental problems per service month | 0.0 (0.0-0.0) | 0.0 (0.0-0.0) | 0.0 (0.0-0.0) | 0.0 (0.0-0.0) |
| Number of visits due to musculoskeletal problems per service month | 0.0 (0.0-0.2) | 0.1 (0.0-0.3) | 0.2 (0.0-0.5) | 0.1 (0.0-0.3) |
|  |  |  |  |  |
| **Did not complete the military service** | **n=24** | **n=26** | **n=61** | **n=111** |
| Number of any garrison visits per service month | 0.6 (0.02-2.8) | 1.3 (0.0-2.0) | 3.0 (1.2-4.2) | 1.9 (0.5-3.6) |
| Number of visits due to mental problems per service month | 0.0 (0.0-0.0) | 0.2 (0.0-1.3) | 0.5 (0.0-1.6) | 0.1 (0.0,1.1) |
| Number of visits due to musculoskeletal problems per service month | 0.0 (0.0-0.9) | 0.0 (0.0-0.8) | 0.0 (0.0-1.5) | 0.1 (0.0-0.5) |
| **Completed the military service** | **n=563** | **n=732** | **n=663** | **n=1958** |
| Number of any garrison visits per service month | 0.3 (0.01-0.6) | 0.4 (0.2-0.8) | 0.6 (0.3-1.2) | 0.5 (0.2-0.9) |
| Number of visits due to mental problems per service month | 0.0 (0.0-0.0) | 0.0 (0.0-0.0) | 0.0 (0.0-0.0) | 0.0 (0.0-0.0) |
| Number of visits due to musculoskeletal problems per service month | 0.0 (0.0-0.2) | 0.1 (0.0-0.3) | 0.2 (0.0-0.5) | 0.1 (0.0-0.3) |
| **All-cause disability retirement** | **n=79** | **n=45** | **n=16** | **n=140** |
| Number of any garrison visits per service month | 0.5 (0.2-0.8) | 0.5 (0.3-1.1) | 2.8 (1.0-4.5) | 0.5 (0.3-1.1) |
| Number of visits due to mental problems per service month | 0.0 (0.0-0.0) | 0.0 (0.0-0.0) | 0.3 (0.0-0.7) | 0.0 (0.0-0.0) |
| Number of visits due to musculoskeletal problems per service month | 0.1 (0.0-0.3) | 0.1 (0.0-0.3) | 0.4 (0.05-1.6) | 0.1 (0.0-0.3) |
| **Did not retire** | **n=508** | **n=713** | **n=708** | **n=1929** |
| Number of any garrison visits per service month | 0.3 (0.1-0.5) | 0.4 (0.2-0.8) | 0.6 (0.3-1.3) | 0.5 (0.2-0.9) |
| Number of visits due to mental problems per service month | 0.0 (0.0-0.0) | 0.0 (0.0-0.0) | 0.0 (0.0-0.0) | 0.0 (0.0-0.0) |
| Number of visits due to musculoskeletal problems per service month | 0.0 (0.0-0.1) | 0.1 (0.0-0.3) | 0.2 (0.0-0.5) | 0.1 (0.0-0.3) |

M, median; IQR, interquartile range
